# The effects of sample size on population genomic analyses – implications for the tests of neutrality

**DOI:** 10.1186/s12864-016-2441-8

**Published:** 2016-02-20

**Authors:** Sankar Subramanian

**Affiliations:** Research Centre for Human Evolution, Environmental Futures Research Institute, Griffith University, 170 Kessels Road, Nathan, Qld 4111 Australia

**Keywords:** Sample size effect, Number of segregating sites, Tests of neutrality, Human genomes and selection

## Abstract

**Background:**

One of the fundamental measures of molecular genetic variation is the Watterson’s estimator (*θ*), which is based on the number of segregating sites. The estimation of *θ* is unbiased only under neutrality and constant population growth. It is well known that the estimation of *θ* is biased when these assumptions are violated. However, the effects of sample size in modulating the bias was not well appreciated.

**Results:**

We examined this issue in detail based on large-scale exome data and robust simulations. Our investigation revealed that sample size appreciably influences *θ* estimation and this effect was much higher for constrained genomic regions than that of neutral regions. For instance, *θ* estimated for synonymous sites using 512 human exomes was 1.9 times higher than that obtained using 16 exomes. However, this difference was 2.5 times for the nonsynonymous sites of the same data. We observed a positive correlation between the rate of increase in *θ* estimates (with respect to the sample size) and the magnitude of selection pressure. For example, *θ* estimated for the nonsynonymous sites of highly constrained genes (*dN*/*dS* < 0.1) using 512 exomes was 3.6 times higher than that estimated using 16 exomes. In contrast this difference was only 2 times for the less constrained genes (*dN*/*dS* > 0.9).

**Conclusions:**

The results of this study reveal the extent of underestimation owing to small sample sizes and thus emphasize the importance of sample size in estimating a number of population genomic parameters. Our results have serious implications for neutrality tests such as Tajima *D*, Fu-Li *D* and those based on the McDonald and Kreitman test: Neutrality Index and the fraction of adaptive substitutions. For instance, use of 16 exomes produced 2.4 times higher proportion of adaptive substitutions compared to that obtained using 512 exomes (24 % vs 10 %).

**Electronic supplementary material:**

The online version of this article (doi:10.1186/s12864-016-2441-8) contains supplementary material, which is available to authorized users.

## Background

Measuring genetic variation is fundamental in population genetics. Molecular genetic variation (*θ*) could be measured as the product of mutation rate (*μ*) and population size (*N*_*e*_) and the theoretical relationship is *θ =* 4*N*_*e*_*μ* (for diploid organisms). Empirically *θ* could be estimated by the Watterson’s estimator *θ*_*w*_ [1] (or simply *θ* hereafter), which is based on the number of segregating sites (*S*) or by the Tajima’s estimator *θ*_*π*_ [2] (*π* hereafter), which uses the mean pair-wise differences between sequences. The estimation of *θ* is based on population coalescent theory and its popularity is due to its simplicity. Hence it is widely used in theoretical and empirical population genetic analyses. For instance *θ* or *S* is used to model the expected number of mutations, which is fundamental in molecular evolutionary biology [3]. *θ* is an unbiased estimator when the assumptions such as neutrality and constant population sizes are met [4]. However, *θ* is downwardly biased for an exponentially growing population. Similarly, estimates of *θ* are biased for sequences under purifying selection, which results in an excess of low frequency variants. This is because the theoretical relationship (*θ =* 4*N*_*e*_*μ*) assumes that all mutations neutral (or observable), which is not true in reality. Therefore this relationship can be written as *θ =* 4*N*_*e*_*μf*, where *f* is the fraction of neutral mutations and *f* = 1 and *f* < 1 for neutral and for selectively constrained sites respectively [5].

Although the factors influencing *θ* are well known, the effects of sample size in modulating the bias in *θ* estimation is not clear. In the pre-genomic era the sample sizes used in population genetics and molecular evolutionary analyses were modest. Therefore, the effects of sample size on fundamental parameters were not well appreciated as the magnitude of these effects were not obvious due to the small differences between the sample sizes used in various studies. However, in the post-genomic period the use of thousands of samples compared to the few dozens of the past will indeed make a huge impact on the estimates [6–9]. Recent studies based on several thousand human exomes identified a huge difference in the *θ* estimates. Nelson et al. [8] studied this issue and compared the *θ* estimated using different sample sizes. They reported that *θ* estimated using the protein-coding genes from 11,000 humans was 4.6 times higher than that estimated using 23 humans (40.4 *vs.* 8.8). In contrast π estimated for the two datasets were identical (3.96). Similarly, another study using exomes from >5,000 Americans showed a negative correlation between sample size and Tajima *D* estimates and up to fourfold difference between the estimates obtained using various sample sizes [9]. Since Tajima *D* is based on the difference between *θ* and *π* the fourfold difference observed in this study was due to the difference in the *θ* estimated for various sample sizes. Similar discrepancies owing to sample size bias were also reported in other organisms such as plants [10]. These studies attributed this phenomenon to the exponential growth of the populations [9]. However, a proper simulation study is needed to confirm this.

Another important factor that is known to bias *θ* estimation is purifying selection. However, whether sample size will modulate the magnitude of this bias is not known. This is an important issue because every gene (and the genome) consists of regions under selection as well as under neutral evolution and most of the population genetic parameters are estimated for both regions. If sample size differentially influences the *θ* estimates of neutral regions and selected regions, then the estimates obtained for these regions are not comparable. This has serious implications for the tests of neutrality such as Tajima *D* [2], Fu and Li *D* [11] and the statistics based on McDonald and Krietman test [12] namely the Neutrality Index (*NI*) [13] and the proportion of adaptive substitutions (*α*) [14].

To examine the differential effects of sample size bias in neutral and constrained sites we assembled a large dataset consisting over 1000 exomes [obtained from the 1000 genomes project [15] and estimated various population genetic parameters. We also examined how and to what extent sample size effects influence the tests of neutrality. Finally, we conducted robust simulations to further elucidate the magnitude of sample size effects on the estimation of *θ* and to determine the probable cause for this pattern.

## Results

### Differential effects of sample size on neutral and selected sites

**Fig. 1:**
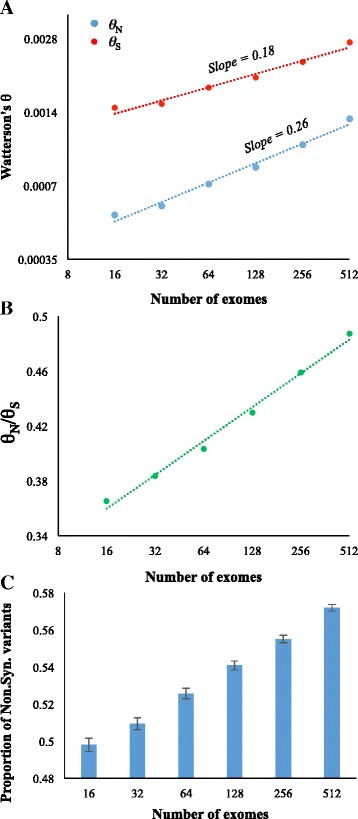
Differential effects of sample size on the estimation of *θ* using the number of synonymous (*θ*_*S*_) and nonsynonymous (*θ*_*N*_) segregating sites. **a** Relationship between the sample size and *θ*. The relationships were significant (*P* < 0.01) based on the Pearson correlation as well as the non-parametric Kendal rank correlation using the log-transformed values of X and Y data points. Best fitting regression lines are shown. X and Y axes are in log-log scales (base 2). **b** Correlation between the number of exomes and the ratio of *θ*_*S*_/*θ*_*N*_. The relationship was statistically significant (*P* < 0.01). **c** The fraction of nonsynonymous variants observed using various sample sizes of human exomes. The error bars denote the standard error estimated using the binomial variance

To examine the effect of sample size on the estimation of *θ* we used 1008 human exomes and grouped them into six categories, each containing non-overlapping 16, 32, 64, 128, 256 and 512 exomes respectively. We then estimated *θ* at synonymous (*θ*_*S*_) and nonsynonymous (*θ*_*N*_) sites of >13000 protein-coding genes. Figure 1a clearly shows that *θ* estimates significantly correlate (*P* < 0.01) and systematically increase with the sample size for both neutral and constrained sites. However, the rate of increase is much higher for constrained than that of neutral sites. This is evident since the slope of the regression line of the former was 44 % higher than that of the latter (0.26 vs 0.18). To further confirm this we estimated the ratio of *θ*_N_/*θ*_S_, which showed a highly significant positive correlation (*P* < 0.01) with the sample size (Fig. 1b). The *θ*_N_/*θ*_S_ estimated for the sample size of 512 was 0.49, which was 32 % higher than that estimated for the size of 16 (0.37). The above results suggest that increase in the sample size lead to the identification of more nonsynonymous variants compared to synonymous SNVs. To confirm this, we estimated the proportion of nonsynonymous SNVs (nSNVs) in each dataset and found positive correlation with the sample size (Fig. 1c). We also repeated our analysis by creating the non-overlapping six-category dataset multiple times through randomly choosing the sequences and obtained consistent results. This was to avoid any bias due to a specific set of exomes in a sample size category.

### Magnitude of purifying selection and the extent of sample size bias

**Fig. 2:**
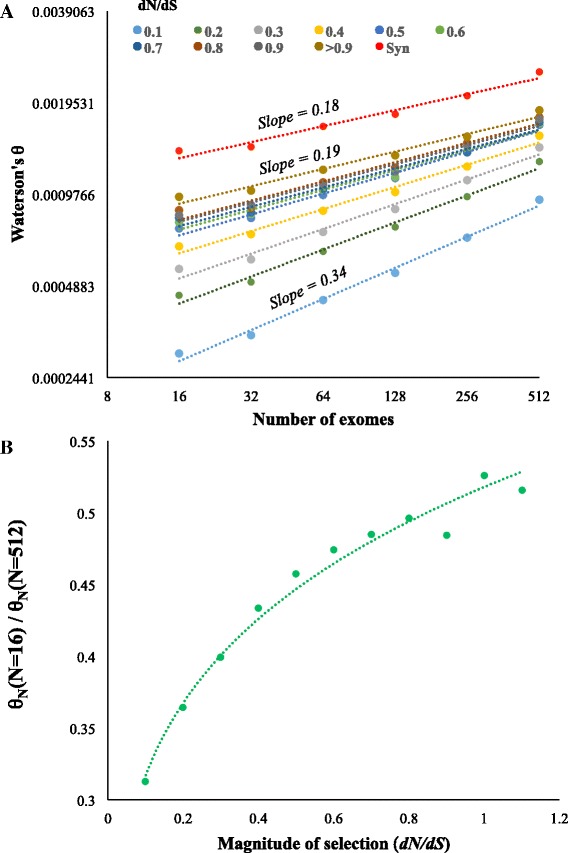
The magnitude of selection pressure and the extent of bias in estimating *θ*. **a** Relationship between sample size and *θ* estimates using the nonsynonymous sites (*θ*_*N*_) of genes under different magnitude of selection. The ratio of nonsynonymous to synonymous substitutions (*dN*/*dS*) was used as a proxy for selection intensity on genes. All relationships were significant (*P* < 0.01). **b** Correlation between the extent of purifying selection (*dN*/*dS*) and the ratio of *θ*_*N*_ estimated using small (*N* = 16) and large (*N* = 512) sample sizes. The relationship was statistically significant (*P* < 0.01)

The above results indicate that the purifying selection on constrained sites could inflate the sample size bias in estimating *θ*. To investigate this further, we grouped the genes based on the magnitude of selection pressure on them. For this purpose, we estimated the *dN*/*dS* ratio for each protein-coding gene and used this as a proxy for the magnitude of selective constraint on them. We then estimated *θ*_N_ for the sets of genes with different *dN*/*dS* ratios (or under varying levels of selection pressure). Fig. 2a shows that with the rate of increase in the estimation of *θ*_N_ (with respect to the sample size) was much higher for the constrained genes compared to those under relaxed selective constraints. For example, the slope of the regression line for the genes with *dN*/*dS* < 0.1 was 0.34, which is 79 % higher than that observed for the genes with *dN*/*dS* > 0.9 (0.19). Note that the slope of the latter was close that observed for the neutral synonymous sites (0.18). To further support these results we computed the ratio between *θ*_N_ estimated using 16 exomes and that estimated using 512 exomes (*θ*_N(16)_/*θ*_N(512)_) (Fig. 2b). We show that these ratios perfectly correspond with the magnitude of selective constraints (*dN*/*dS*) on the genes (*P* < 0.01).

### Sample size effects on Tajima *D* (*D*_*T*_) and Fu-Li *D* (D_*FL*_)

**Fig. 3:**
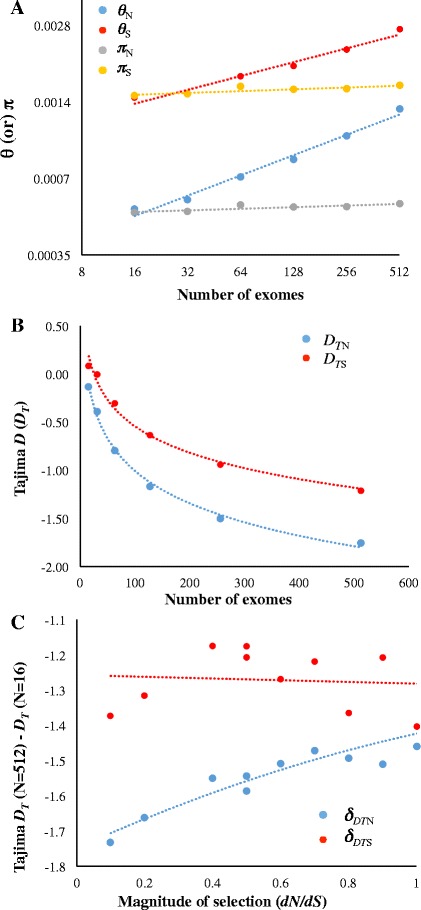
Sample size effects on Tajima *D*_*T*_ statistics. **a** *θ* and *π* were estimated for synonymous (*θ*_*S*_ and *π*_*S*_) and nonsynonymous sites (*θ*_*N*_ and *π*_*N*_) using different sample sizes. While relationships between sample size and *θ* was significant (*P* < 0.01) those between sample size and *θ* were not (*P* = 0.06). **b** Scatter plot shows the Tajima *D*_*T*_ estimated for nonsynonymous (*D*_*TN*_) and synonymous sites (*D*_*TS*_) using different sample sizes of human exomes. The relationships were significant at the 1 % level. **c** Relationship between the magnitude of selection pressure on protein-coding genes (*dN*/*dS*) and the difference (*δ*_*DT*_) in Tajima *D*_*T*_ estimated using large (*N* = 512) and small (*N* = 16) number of exomes. The plot shows the comparative patterns of the significant relationships observed for neutral (*δ*_*DTS*_) and constrained (*δ*_*DTN*_) sites. Each data point indicates the *δ*_*DTS*_ or *δ*_*DTN*_ estimated using the genes belong to a selection intensity category (eg. *dN*/*dS* < 0.1). The correlation involving constrained sites was significant (*P* < 0.01) but that of neutral sites was not (*P* = 0.48)

Apart from the number of segregating sites another popular measure for the extent of variation is nucleotide diversity (*π*). Unlike *θ* this measure is not affected by population growth or purifying selection. Hence we compared these two measures when they are estimated with various samples sizes. This reveals that *π* is not affected by sample sizes not only for neutral sites (*P* > 0.05) but also for constrained sites (*P* > 0.05) (Fig. 3a). The popular Tajima *D* (*D*_*T*_) statistic uses the properties of *π* and *θ* to test a genomic region for any deviation from neutral evolution. As we have shown that sample size influences only one of these two measures (*θ*) we examined the extent of its effect on *D*_*T*_. As expected, we observed negative correlation (*P* < 0.01) between *D*_*T*_ and sample size for neutral and constrained sites (Fig. 3b). However, the extent of this overestimation was much higher for constrained than neutral sites. This is evident from the widening of the *D*_*T*_ estimates of constrained (*D*_*TN*_) and neutral sites (*D*_*TS*_) with increasing sample size. For instance, the difference between *D*_*TN*_ and *D*_*TS*_ was only 0.22 for a sample size of 16. But this value was 0.54 for a sample size of 512, which is more than two fold higher than the former. We also examined the relationship between the magnitude of selection and extent of bias in estimating *D*_*TN*_. For this purpose, we estimated *D*_*TN*_ using the genes under varying levels of selection pressures. We computed *D*_*TN*_ for the synonymous and nonsynonymous sites of these genes. We then measured the difference (*δ*_*DTN*_) between the *D*_*TN*_ value obtained for the lowest (*N* = 16) and highest (*N* = 512) sample sizes. This measure *δ*_*DTN*_ was computed for 10 gene sets with *dN*/*dS* ranging between 0–1 with an interval of 0.1 (Fig. 3c). We observed a significant positive correlation (*P* < 0.001) between *dN*/*dS* and *δ*_*DTN*_ for the nonsynoymous sites of protein-coding gene sets. In contrast this relationship was not observed for the synonymous sites of the same gene sets (*P* = 0.48). This suggests that the level of selective constraint significantly influences the estimation *D*_*TN*_ of different genes. On the contrary the bias in the estimation of *D*_*TS*_ is similar across the genes under various levels of selection pressures.

**Fig. 4:**
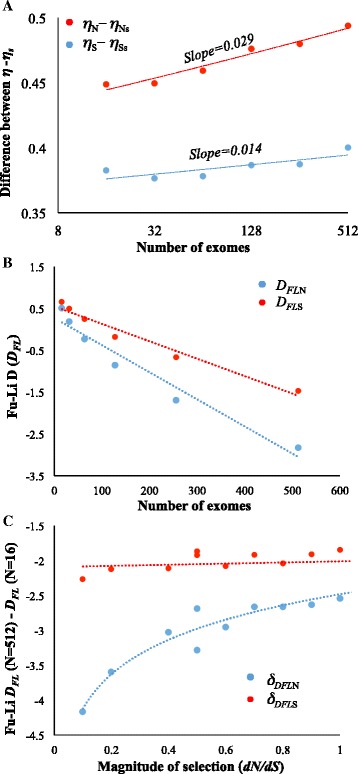
Influence of sample size on the estimation of Fu-Li *D*_*FL*_. **a** The difference between the total number of mutations (*η*) and singleton mutations (*η*_*s*_) estimated for neutral (*η*_*S*_ and *η*_*Ss*_) and constrained (*η*_*N*_ and *η*_*Ns*_) sites using various numbers of human exomes. **b** Scatter plot shows Fu and Li *D* estimated for nonsynonymous (*D*_*FLN*_) and synonymous sites (*D*_*FLS*_) using different sample sizes. The relationships were significant at the 1 % level. **c** Relationship between the magnitude of selection pressure on protein-coding genes (*dN*/*dS*) and the difference (*δ*_*DFL*_) in Fu-Li *D*_*FL*_ estimated using large (*N* = 512) and small (*N* = 16) number of exomes. The plot shows the comparative patterns of the relationships observed for neutral (*δ*_*DFLS*_) and constrained (*δ*_*DFLN*_) sites. Each data point indicates the *δ*_*DFLS*_ or *δ*_*DFLN*_ estimated using the genes belong to a selection intensity category (eg. *dN*/*dS* = 0.1-0.2). The correlation involving constrained sites was significant (*P* < 0.01) but that of neutral sites was not (*P* = 0.14)

We then examined the effect of sample size on the other popular test of neutrality, the Fu-Li *D* test - without using an outgroup (*D*_*FL*_*)*. This test uses the difference between the total number of mutations (*η*) and the singleton (appearing only once in the genealogy) mutations (*η*_*s*_). We computed the difference (*η* – *η*_*s*_) for synonymous and nonsynonymous sites of protein-coding genes using different sample sizes. While the difference for neutral sites (*η*_*S*_ – *η*_*Ss*_) only slightly varied with sample sizes that (*η*_*N*_ – *η*_*Ns*_) for constrained sites significantly increased with sample size (Fig. 4a). The slope of the regression line for the latter was (0.029) higher than that observed for the former (0.014). This result clearly predicts differential effects of sample size on *D*_*FL*_ as well. To confirm this, we estimated *D*_*FL*_ for synonymous (*D*_*FLS*_) and nonsynonymous sites (*D*_*FLN*_) using different sample sizes. We found a positive relationship between *D*_*FL*_ and sample size (*P* < 0.01) and the bias in the estimation of *D*_*FL*_ was more pronounced for the constrained sites (Fig. 4b) than neutral sites. The *D*_*FLN*_ estimates obtained using a sample sizes of 512 was 6 times higher than that estimated using the sample size of 16. In contrast this difference was only 3 times for *D*_*FLS*_. We also observed a positive relationship between *dN*/*dS* and *δ*_*DFLN*_ for constrained sites (*P* < 0.01). However, there was no significant relationship between sample size and *δ*_*DFLS*_ for the neutral sites (*P* = 0.14) (Fig. 4c). Hence the patterns observed for Fu-Li *D*_*FL*_ were similar to those detected for Tajima *D*_*T*_, which clearly emphasize the significance of sample size-mediated bias in estimating these parameters.

### Influence of sample size on MK-test based measures

**Fig. 5:**
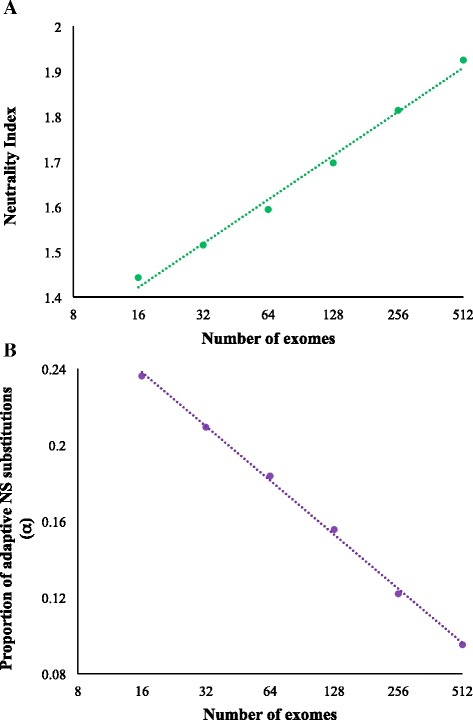
Effect of sample size on the McDonlad and Kreitman test based statistics. **a** Correlation between Neutrality Index and the number of exomes used to estimate this measure. **b** The proportion of adaptive nonsynonymous substitution estimated using different sample sizes. Both the relationships were statistically significant at the 1 % level

Two routinely used population genetic measures that are based on the principles of McDonald and Kreitman test are the Neutrality Index and the fraction of adaptive nonsynonymous substitutions (*α*). Both these measures use polymorphisms and substitutions in synonymous and nonsynonymous positions. To examine the effect of sample size on these parameters we computed them using different numbers of human exomes. We observed a highly significant positive relationship (*P* < 0.01) between sample size and Neutrality Index (*NI*) (Fig. 5a). The values of *NI* for the sample sizes of 16 and 512 were 1.4 and 1.9 respectively showing a 36 % difference. Since *NI* is the measure of purifying selection using a smaller sample size significantly underestimates the selection pressure on human exomes.

Finally, we quantified the proportion of adaptive nonsynonymous substitutions (*α*) using different sample sizes. Our result produced a strong negative correlation (*P* < 0.01) between the sample size and *α* (Fig. 5b). Interestingly using a small number of exomes suggested that 24 % of the nonsynonymous mutations were fixed by adaptive evolution. However, using a much larger sample size of 512 exomes this number reduced 2.4 fold and only 10 % of the nonsynonymous substitutions were estimated to be under positive selection.

### Results from simulation analysis

**Fig. 6:**
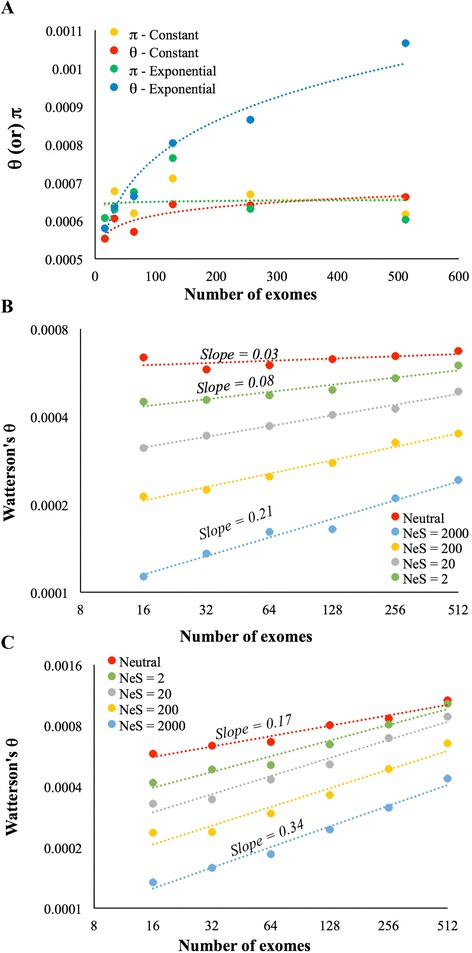
Results from the simulation study. **a** Estimation of *θ* and *π* using different number of genome sequences simulated under neutral evolution and constant as well as exponential growth models. Only the relationship between sample size and *θ* estimated for the exponential growth model was significant (*P* < 0.01) and other three were not (*P* > 0.06). **b** The estimates of *θ* obtained for the sequences simulated under neutral and under different levels of purifying selection using constant population growth model. The relationship between sample size and *θ* estimated for the sequences simulated under neutral evolution was not significant (*P* = 0.13). All other relationships were significant (*P* < 0.01). **c** *θ* estimates for the sequences simulated under neutral and under different levels of purifying selection using exponential population growth model. The figure shows the correlation between *θ* estimates and the number of simulated sequences used for the estimation. All relationships were significant at the 1 % level

To investigate whether *θ* estimated for exponentially growing populations and for selectively constrained genomic regions is seriously influenced by sample size we conducted a simulation study using the program SFS_CODE [16]. We modelled two populations that undergo a similar initial growth phases, but one was later allowed to grow exponentially and the other remained under constant population growth (see Methods). We also repeated this model for neutrally evolving sequences as well as for those under different levels of selection pressures. Both simulations were conducted for varying sample sizes of 16, 32, 64, 128, 256 and 512. The results from the sequences simulated under neutrality clearly show that the *θ* estimated for the population under constant population growth did not vary with sample sizes (*P* > 0.05) (Fig. 6a). On the contrary *θ* estimated for the sequences simulated under exponential growth significantly correlated with sample size (*P* < 0.01) and these estimates varied up to 84 % between the samples sizes of 16 and 512. However, the estimates of *π* were not affected by sample sizes and this is true for the populations under constant as well as exponential growth (*P* > 0.05).

The results from the sequences simulated under varying levels of selective constraints are shown in Figs. 6b and c for constant and exponential growth models respectively. Figure 6b shows that even under constant population growth conditions the estimation of *θ* varied with sample sizes. The rate of variation was much higher for sequences under high selective constraints (*NeS* = 2000) than those under relaxed selective pressures (*NeS* = 2). The slope of the regression line of the former was 2.7 times higher than that of the latter (0.21 Vs 0.08). However, *θ* estimated for neutrally evolving sequences did not vary with the sample size as there was no significant correlation between the two variables (*P* < 0.13). These results suggest that for populations under constant growth, purifying selection alone modulate the sample size bias in estimating *θ*.

Our simulation results for exponentially growing population shows that the sample size bias in estimating *θ* is much higher compared to that under constant growth. For instance, the slopes of the regression lines shown in Fig. 6c are much higher (0.17 – 0.34) than the corresponding lines shown in Fig. 6b (0.03 – 0.21). Furthermore, the difference in *θ* estimates obtained for large (*N* = 512) and small (*N* = 16) sample sizes are also much higher for exponentially growing populations than those under constant growth. For instance, this difference was 3.3 times for the highly constrained (*NeS* = 2000) exponentially growing populations (blue circles-Fig. 6c) and this was only 2.1 times for those under constant growth (blue circles-Fig. 6b). The overall results from our simulation study were qualitatively identical to those observed using the 1000 genome human data.

## Discussion

**Fig. 7:**
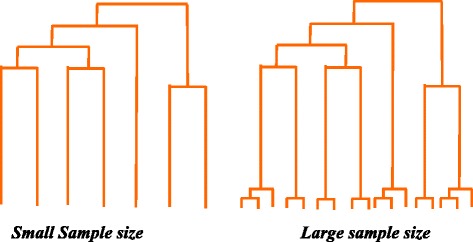
Hypothetical trees show the effects of sample size. Large sample size increases the resolution that lead to the detection of rare and personal variants shown in the tips of the tree

In this study we showed that there is an effect of sample size in estimating the fundamental population genetic parameter, *θ*. Previous studies based on human exome data reported the sample size effect on the estimation of *θ* and attributed this to the exponential mode of growth in human populations [7–10, 17]. In this study we showed that the sample size effect could also be significantly modulated by purifying selection. The results from the simulation study clearly highlighted the independent contributions of demography (exponential growth) and selection in influencing *θ* estimation for different sample sizes. The patterns of our observed and simulated results could be explained based on the fact that the resolution in identifying low frequency variants increases with the increase in the sample size. When a population undergoes constant growth phase, the distribution of SNVs (site frequency distribution) follows standard coalescence model and Watterson’s *θ* clearly captures this. However, when a population grows exponentially, a higher proportion of low frequency variants are created due to faster coalescence events near the tip of the genealogies. This is shown in Fig. 7 and the hypothetical population tree based on large sample size has more branches in the tips suggesting more observable mutations when the sample size is large [9]. Hence for an exponentially growing population a higher *θ* is expected. However, to observe or to identify the vast majority of low frequency variants a larger sample size is required. In the context of mutations, small sample size underestimates the overall mutations in a population as it misses rare mutations and a higher sample size is needed to observe all of them.

The role of selection could be explained by comparing the regions under neutrality to those under selective constraints. Since purifying selection prevents deleterious mutations reaching high frequencies, constrained genomic regions are typically abundant in low frequency SNVs [18]. Therefore, large sample sizes are required to properly identify these rare SNVs. Hence the estimation of *θ* for the constrained regions of exponentially growing populations is much more severely biased by sample sizes because they are modulated by both demographic *and* selective forces. Since the human population is known to be under exponential growth, the sample size effects on the estimation of *θ* for neutral synonymous sites are influenced by the demographic factor alone but estimates for the nonsynonymous sites are modulated by both demographic and selective forces. This is clear in the simulation study, which showed that the sample size bias is influenced only by purifying selection in constant populations. Therefore, the extent of sample size bias for the constrained regions of exponentially growing populations was much higher than that observed for the constrained regions of populations under constant growth. Furthermore, humans have a unique demographic history and it is well known that human populations have undergone an explosive population growth, which resulted in much higher fraction of rare deleterious variants [8, 9]. This is evident from the unusually high ratio of *θ*_N_/*θ*_S_ (0.5) observed for large sample sizes (Fig. 1b).

Apart from neutrality and constant growth, the estimation of *θ* is also based on the assumption that individual sites/mutations in a genome are inherited and evolve independently. However, this is not true in reality as the genomic regions along with the mutations are inherited as large blocks of IBD segments. Therefore, the equation used for *θ* estimation does not account for this and therefore the results shown in this study might have the influence of this bias.

In this study we have used the data 1000 genome project, which consists of genomes from a number of populations all over the world. Hence this sample composition is from a continuous population, which is a unique and unusual characteristic of this dataset. Therefore, to examine the generality of the patterns observed in this study for specific populations we examined the sample size issue using single populations. For this purpose, we used the subset of 85 exomes belonging to the CEU (Utah American) population and divided the data into two groups, one with small (16 exomes) and another with large (64 exomes) sample sizes (Additional file 1: Figure S1). For neutral genomic regions (synonymous sites) the *θ* estimate obtained for the large sample size was 9 % higher than that observed for small sample sizes and the difference was highly significant (*P* < 10^−7^). In contrast for constrained regions this difference was 26 % (*P* < 10^−7^). As expected, *π* estimates were similar between large and small sample sizes and this was true for neutral (*P* = 0.48) as well as constrained sites (*P* = 0.61). Similar results were observed for African (YRI) and Asian (CHB) populations (Additional file 1: Figures S2 and S3). Although we could perform the population specific analyses using only a small number of available exomes the results were highly significant and qualitatively similar to the main results reported in this study.

## Conclusions

The results of this study highlight the significance of sample size in estimating some of the fundamental parameters of population genetics. Importantly we showed that for small sample sizes the underestimation of *θ* is higher for constrained regions than that for neutral regions of the same set of exomes. Hence the different *θ* estimated for the two regions using same population genomic data are not comparable especially when the sample size is small. Therefore, this bias affects all neutrality tests and the estimates based on them. For instance, Fig. 3 shows that the difference in Tajima’s *D* estimated for neutral and constrained sites widens with the increase in sample size. In fact, the (close to) true values will only be obtained for very large sample sizes. When sample size is small Tajima’s *D* of the two types of sites are apparently similar. Hence use of small number of samples in this analysis will produce erroneous results due to severe underestimation of *θ* for constrained sites. This also is true for the results of Fu-Li D test (Fig. 4).

In the case of MK test based statistics, the proportion of adaptive nonsynonymous substitutions (*α*), use of large number of samples results in identifying more deleterious (low frequency) nonsynonymous SNVs, which increases *P*_*n*_ in eqn 5 and thus the value of *α* is reduced. In contrast, small sample sizes identify fewer nonsynonymous SNVs, which leads to an overestimation of the proportion of adaptive substitutions. The other measure based on MK-test, the Neutrality Index, is underestimated using a small number of samples due to the failure to precisely identify some of the low frequency nonsynonymous SNVs.

## Methods

### Genomic sequence data and analyses

We obtained the genome data for 1092 humans from GenBank, which was originally generated by the 1000 genome project (phase 1-version 3) [15]. Using the genome annotations, we extracted the single nucleotide variants (SNVs) present in the synonymous and nonsynonymous sites of protein-coding genes and included only the bi-allelic SNVs. We divided the data into six non-overlapping sets consisting of 16, 32, 64, 128, 256 and 512 exomes (or samples). To determine the magnitude of selection on nonsynonymous sites we used the *dN*/*dS* ratio computed for the protein-coding genes using the human-chimpanzee pair. For this purpose, we obtained the human-chimpanzee pair-wise genome alignment from the UCSC genome browser data resource (https://genome.ucsc.edu/). Using the exon-intron boundaries provided in the reference gene annotations we extracted the protein-coding transcripts from the human-chimp alignment. Using the gene annotations from *Ensembl* (http://www.ensembl.org/) we retained the longest transcript for each gene. For each gene the divergence at synonymous sites (*dS*) and nonsynonymous sites (*dN*) were estimated based on the maximum likelihood method employed in the software PAML [19]. While *dS* (S_M_/S_S_) is the number of synonymous substitutions (S_M_) per synonymous site (S_S_) in a gene *dN* (N_M_/N_S_) is the number of nonsynonymous substitutions (N_M_) per nonsynonymous site (N_S_). In estimating *dN* or *dS*, the maximum likelihood method tend to overestimate when the actual divergence is large. To avoid such estimation errors (due to the overcorrection of multiple hits) we excluded the genes for which *dN* or *dS* estimate was > 0.8. These filters resulted in 13,454 unique protein-coding genes, which were eventually used for further analysis. The ratio of *dN* and *dS* (*dN*/*dS*) was used as the proxy for the magnitude of selection pressure on a gene.

### Parameter estimation

We estimated a number of population genetic parameters such as *θ*, *π*, Tajima *D*, Fu-Li *D*, Neutrality Index and the proportion of adaptive nonsynonymous substitutions using the following equations.

#### Estimation of *θ* and *π*

The Watterson’s estimator (*θ*) measures the molecular genetic variation as the population scaled mutation rate using the number of segregating sites as [1]:

$$ \theta =\frac{S}{a_n} $$

where *S* is the number of segregating sites, *n* is the number of sequences and ${a}_n = {\displaystyle \sum_{i=1}^{n-1}}\frac{1}{i}$. In this study we estimated *θ* and *S* as the number of segregating sites per site. Nucleotide diversity (*k*) is the average number of pair-wise nucleotide differences between sequences, which was estimated using the following equation [2]:

$$ k=\frac{{\displaystyle \sum_{i<j}{\displaystyle \sum {k}_{ij}}}}{\left(\begin{array}{c}\hfill n\hfill \\ {}\hfill 2\hfill \end{array}\right)} $$

In this study we used *π* rather than *k*, which is the average number of pair-wise nucleotide differences per site.

#### *Tajima D* (*D*_*T*_)

This test is based on the difference between the number of segregating sites and average number of pair-wise nucleotide differences. Under neutrality these two measures are expected to be equal. Tajima’s *D* is given by [2]:

$$ {D}_T=\frac{k - \frac{S}{a_1}}{\sqrt{e_1S+{e}_1S\left(S-1\right)}} $$

where S is the number of segregating sites, *k* is the average number of pair-wise nucleotide differences between sequences and *e*_1_ and *e*_2_ are given by the equations 36 and 37 of Tajima [2].

#### *Fu and Li D* (*D*_*FL*_)

This is another neutrality test similar to Tajima *D*_*T*_ but based on the difference between the total number of mutations and the singleton mutations in a population genealogy. Under neutrality these two numbers are expected to be equal. The *D*_*FL*_ of Fu and Li (without outgroup) is given by [11]:

$$ {D}_{FL} = \frac{\left(\frac{n}{n-1}\right)\ \eta - {a}_n{\eta}_s}{\sqrt{u_D\eta +{v}_D{\eta}^2}} $$

where *η* is the total number of mutations, *η*_*s*_ is the number of singleton mutations in the sequences and *u*_*D*_ and *v*_*D*_ are given by the equations in page 701 of Fu and Li [11]. We used the total number of segregating sites and the number of singleton sites as the proxy for *η* and *η*_*s*_ respectively as suggested [20].

#### *McDonald and Kreitman* (*MK*) *test*

MK test uses the ratio of nonsynonymous to synonymous polymorphisms and divergence [12]. Under neutrality these ratios are expected to be equal as given by:

$$ \frac{P_n}{P_s}=\frac{D_n}{D_s} $$

where *P*_*n*_ and *P*_*s*_ are number of nonsynonymous and synonymous polymorphisms and *D*_*n*_ and *D*_*s*_ are number of nonsynonymous and synonymous substitutions respectively.

The popular statistics used in population genetics namely the Neutrality Index (*NI*) and the proportions of adaptive nonsynonymous substitutions (*α*) are based on the principles of the MK test.

#### Neutrality Index (NI)

*NI* is the ratio of the two ratios, which is given by [13]:

$$ NI = \raisebox{1ex}{$\frac{P_n}{P_s}$}\!\left/ \!\raisebox{-1ex}{$\frac{D_n}{D_s}$}\right. $$

#### Proportion of adaptive nonsynonymous substitutions (*α*)

This measure is routinely used to quantify adaptive nonsynonymous substitutions in protein-coding genes when inter-species as well as within species (population) genomic data are available [14]. *α* can be estimated as:

$$ \alpha =1-\frac{D_s{P}_n}{D_n{P}_s} $$

### Simulation

We conducted an extensive simulation using the program SFS_CODE [16], which is based on forward-in-time population genetic model. The simulation was performed under constant and exponential growth models. Sequences were also simulated for neutral evolution and purifying selection. A sequence length of 5000 bp, *N*_e_ = 10,000 and a mutation rate of 1 × 10^−8^ per site per generation was used for the simulation [21]. We conducted separate simulation runs using sample sizes of 16, 32, 64, 128, 256 and 512. For human population growth we followed the model proposed by Tennessen et al. [9]. This model uses two growth phases, the first one was slow and a second one was exponentially fast. To keep the simulations comparable between constant and exponential growth models we combined the simulation runs and used the parameters suggested by a previous study [22]. In the beginning we simulated a population that first splits into two and both grow at the same rate until they reach *N*_e_ = 9,210. Then only one population undergoes a large exponential growth phase until it reaches *N*_e_ = 512,210. The other population undergoes a constant growth phase and thus its number remains at *N*_e_ = 9,210. For modelling constrained site evolution, we used the scaled selection coefficient *γ* = 2*Ns* with *γ* following a gamma distribution, which has a mean of *α*/*β*. We fixed *α* as 0.206 based on previous studies [23] and varied *β* to model various magnitudes of selection ranging between *γ* = 2 to 2000. We performed 1000 replicates, obtained the estimates *θ* and π and computed the mean values. Since the simulation conducted here was only to compare the *θ* estimates from different sample sizes changing any parameter (eg. mutation rate) does not affect the end results.

## Additional file

Additional file 1: Figure S1. Theta and Pi estimates using 80 CEU (Utah Americans) exomes. The data was divided into large (64) and small (16) sample sizes to estimate theta and pi. (A) Using synonymous sites of protein-coding genes (B) nonsynonymous sites. The error bars denote standard error. We used a bootstrap (1000 replications) procedure to estimate the variance. **Figure S2**. Theta and Pi estimates using 80 CHB (Han Chinese) exomes. The data was divided into large (64) and small (16) sample sizes to estimate theta and pi. (A) Using synonymous sites of protein-coding genes (B) nonsynonymous sites. The error bars denote standard error. We used a bootstrap (1000 replications) procedure to estimate the variance. **Figure S3**. Theta and Pi estimates using 80 YRI (Yoruban) exomes. The data was divided into large (64) and small (16) sample sizes to estimate theta and pi. (A) Using synonymous sites of proteincoding genes (B) nonsynonymous sites. The error bars denote standard error. We used a bootstrap (1000 replications) procedure to estimate the variance. (DOCX 85 kb)

## References

[1] Watterson GA (1975). On the number of segregating sites in genetical models without recombination. Theor Popul Biol.

[2] Tajima F. Statistical method for testing the neutral mutation hypothesis by DNA polymorphism. Genetics. 1989;123(3):585–95. PubMed PMID: 2513255; PubMed Central PMCID: PMCPMC1203831. 10.1093/genetics/123.3.585 PMC1203831 2513255

[3] Sawyer SA, Hartl DL. Population genetics of polymorphism and divergence. Genetics. 1992;132(4):1161–76. PubMed PMID: 1459433; PubMed Central PMCID: PMCPMC1205236. 10.1093/genetics/132.4.1161 PMC1205236 1459433

[4] Nei M, Kumar S (2000). Molecular Evolution and Phylogenetics.

[5] Henn BM, Botigue LR, Bustamante CD, Clark AG, Gravel S (2015). Estimating the mutation load in human genomes. Nat Rev Genet.

[6] Al-Khudhair A, Qiu S, Wyse M, Chowdhury S, Cheng X, Bekbolsynov D, et al. Inference of distant genetic relations in humans using "1000 genomes". Genome Biol Evol. 2015;7(2):481–92. doi:10.1093/gbe/evv003. PubMed PMID: 25573959; PubMed Central PMCID: PMCPMC4350174. 10.1093/gbe/evv003 PMC4350174 25573959

[7] Korneliussen TS, Moltke I, Albrechtsen A, Nielsen R. Calculation of Tajima's D and other neutrality test statistics from low depth next-generation sequencing data. BMC Bioinformatics. 2013;14:289. doi:10.1186/1471-2105-14-289. PubMed PMID: 24088262; PubMed Central PMCID: PMCPMC4015034. 10.1186/1471-2105-14-289 PMC4015034 24088262

[8] Nelson MR, Wegmann D, Ehm MG, Kessner D, St Jean P, Verzilli C (2012). An abundance of rare functional variants in 202 drug target genes sequenced in 14,002 people. Science.

[9] Tennessen JA, Bigham AW, O'Connor TD, Fu W, Kenny EE, Gravel S (2012). Evolution and functional impact of rare coding variation from deep sequencing of human exomes. Science.

[10] Larsson H, Kallman T, Gyllenstrand N, Lascoux M. Distribution of long-range linkage disequilibrium and Tajima's D values in Scandinavian populations of Norway Spruce (Picea abies). G3 (Bethesda). 2013;3(5):795–806. doi:10.1534/g3.112.005462. PubMed PMID: 23550126; PubMed Central PMCID: PMCPMC3656727. 10.1534/g3.112.005462 PMC3656727 23550126

[11] Fu YX, Li WH. Statistical tests of neutrality of mutations. Genetics. 1993;133(3):693–709. PubMed PMID: 8454210; PubMed Central PMCID: PMCPMC1205353. 10.1093/genetics/133.3.693 PMC1205353 8454210

[12] McDonald JH, Kreitman M (1991). Adaptive protein evolution at the Adh locus in Drosophila. Nature.

[13] Rand DM, Kann LM (1996). Excess amino acid polymorphism in mitochondrial DNA: contrasts among genes from Drosophila, mice, and humans. Mol Biol Evol.

[14] Smith NG, Eyre-Walker A (2002). Adaptive protein evolution in Drosophila. Nature.

[15] Abecasis GR, Auton A, Brooks LD, DePristo MA, Durbin RM, et al. An integrated map of genetic variation from 1,092 human genomes. Nature. 2012;491(7422):56–65. doi:10.1038/nature11632. PubMed PMID: 23128226; PubMed Central PMCID: PMCPMC3498066. 10.1038/nature11632 PMC3498066 23128226

[16] Hernandez RD. A flexible forward simulator for populations subject to selection and demography. Bioinformatics. 2008;24(23):2786–7. doi:10.1093/bioinformatics/btn522. PubMed PMID: 18842601; PubMed Central PMCID: PMCPMC2639268. 10.1093/bioinformatics/btn522 PMC2639268 18842601

[17] Zhang Q, Tyler-Smith C, Long Q. An extended Tajima's D neutrality test incorporating SNP calling and imputation uncertainties. Stat Interface. 2015;8(4):447–56. doi:10.4310/SII.2015.v8.n4.a4. PubMed PMID: 26681995; PubMed Central PMCID: PMCPMC4678577. 10.4310/SII.2015.v8.n4.a4 PMC4678577 26681995

[18] Subramanian S. The abundance of deleterious polymorphisms in humans. Genetics. 2012;190(4):1579–83. doi:10.1534/genetics.111.137893. PubMed PMID: 22267501; PubMed Central PMCID: PMCPMC3316666. 10.1534/genetics.111.137893 PMC3316666 22267501

[19] Yang Z (2007). PAML 4: phylogenetic analysis by maximum likelihood. Mol Biol Evol.

[20] Li W-H (1997). Molecular Evolution.

[21] Roach JC, Glusman G, Smit AF, Huff CD, Hubley R, Shannon PT, et al. Analysis of genetic inheritance in a family quartet by whole-genome sequencing. Science. 2010;328(5978):636–9. doi:10.1126/science.1186802. PubMed PMID: 20220176; PubMed Central PMCID: PMCPMC3037280. 10.1126/science.1186802 PMC3037280 20220176

[22] Gazave E, Chang D, Clark AG, Keinan A (2013). Population growth inflates the per-individual number of deleterious mutations and reduces their mean effect. Genetics.

[23] Boyko AR, Williamson SH, Indap AR, Degenhardt JD, Hernandez RD, Lohmueller KE (2008). Assessing the evolutionary impact of amino acid mutations in the human genome. PLoS genetics.

