# Supplementary material for: The effects of sample size on population genomic analyses – implications for the tests of neutrality
Source: BMC Genomics. 2016 Feb 20;17:123. doi: 10.1186/s12864-016-2441-8 (PMC4761153; doi:10.1186/s12864-016-2441-8)
Supplement: Additional file 1: Figure S1. — Theta and Pi estimates using 80 CEU (Utah Americans) exomes. The data was divided into large (64) and small (16) sample sizes to estimate theta and pi. (A) Using synonymous sites of protein-coding genes (B) nonsynonymous sites. The error bars denote standard error. We used a bootstrap (1000 replications) procedure to estimate the variance. Figure S2. Theta and Pi estimates using 80 CHB (Han Chinese) exomes. The data was divided into large (64) and small (16) sample sizes to estimate theta and pi. (A) Using synonymous sites of protein-coding genes (B) nonsynonymous sites. The error bars denote standard error. We used a bootstrap (1000 replications) procedure to estimate the variance. Figure S3. Theta and Pi estimates using 80 YRI (Yoruban) exomes. The data was divided into large (64) and small (16) sample sizes to estimate theta and pi. (A) Using synonymous sites of proteincoding genes (B) nonsynonymous sites. The error bars denote standard error. We used a bootstrap (1000 replications) procedure to estimate the variance. (DOCX 85 kb) [file 12864_2016_2441_MOESM1_ESM.docx]

**Figure S1.** Theta and Pi estimates using 80 CEU (Utah Americans) exomes. The data was divided into large (64) and small (16) sample sizes to estimate theta and pi. (A) Using synonymous sites of protein-coding genes (B) nonsynonymous sites. The error bars denote standard error. We used a bootstrap (1000 replications) procedure to estimate the variance.

**Figure S2.** Theta and Pi estimates using 80 CHB (Han Chinese) exomes. The data was divided into large (64) and small (16) sample sizes to estimate theta and pi. (A) Using synonymous sites of protein-coding genes (B) nonsynonymous sites. The error bars denote standard error. We used a bootstrap (1000 replications) procedure to estimate the variance.

**Figure S3.** Theta and Pi estimates using 80 YRI (Yoruban) exomes. The data was divided into large (64) and small (16) sample sizes to estimate theta and pi. (A) Using synonymous sites of protein-coding genes (B) nonsynonymous sites. The error bars denote standard error. We used a bootstrap (1000 replications) procedure to estimate the variance.
